# Room-Temperature Synthesis of Air-Stable Near-Infrared Emission in FAPbI_3_ Nanoparticles Embedded in Silica

**DOI:** 10.3390/bios11110440

**Published:** 2021-11-04

**Authors:** Lung-Chien Chen, Li-Wei Chao, Chen-Yu Xu, Chih-Hung Hsu, Yi-Ting Lee, Zi-Min Xu, Chun-Cheng Lin, Zong-Liang Tseng

**Affiliations:** 1 Department of Electro-Optical Engineering, National Taipei University of Technology, Taipei 106344, Taiwan; ocean@ntut.edu.tw (L.-C.C.); aa0932693328@gmail.com (L.-W.C.); eok26732687@gmail.com (Z.-M.X.); 2 Department of Electronic Engineering, Ming Chi University of Technology, New Taipei City 24301, Taiwan; u06157020@mail2.mcut.edu.tw; 3 Giant-Tek Corporation, Miaoli County 35048, Taiwan; rex@giant-tex.com.tw; 4 Center for Organic Photonics and Electronics Research (OPERA), Kyushu University, 744 Motooka, Nishi, Fukuoka 819-0395, Japan; ytlee@opera.kyushu-u.ac.jp; 5 Department of Mathematic and Physical Sciences, General Education, R.O.C. Air Force Academy, Kaohsiung 82047, Taiwan

**Keywords:** perovskite, nanocrystals, APTES, CH(NH_2_)_2_PbI_3_, FAPbI_3_, NIR

## Abstract

Hybrid organic−inorganic and all-inorganic metal halide perovskite nanoparticles (PNPs) have shown their excellent characteristics for optoelectronic applications. We report an atmospheric process to embed formamidinium CH(NH_2_)_2_PbI_3_ (FAPbI_3_) PNPs in silica protective layer at room temperature (approximately 26 °C) employing (3-aminopropyl) triethoxysilane (APTES). The resulting perovskite nanocomposite (PNCs) achieved a high photoluminescence (PL) quantum yield of 58.0% and good stability under atmospheric moisture conditions. Moreover, the PNCs showed high PL intensity over 1 month of storage (approximately 26 °C) and more than 380 min of PNCs solutions in DI water. The studied near-infrared (NIR) light-emitting diode (LED) combined a NIR-emitting PNCs coating and a blue InGaN-based chip that exhibited a 788 nm electroluminescence spectrum of NIR-LEDs under 2.6 V. This may be a powerful tool to track of muscle and disabled patients in the detection of a blood vessel.

## 1. Introduction

Organic–inorganic Formamidinium lead halide (CH(NH_2_)_2_PbX_3_ or FAPbX_3_, X = Cl, Br, I) perovskite nanoparticles (PNPs) have been regarded as novel materials for many optoelectronic applications owing to their advanced class of direct bandgap and excellent photophysical properties, such as strong absorption coefficient, narrow emission width, ease of size control, and so on [1,2,3,4,5,6,7]. The applications of such PNPs have also been shown in different fields, including solar cells [8], sensitive photodetectors [9,10,11,12,13], low threshold lasers [14,15], laser diodes [16], and light emitting diodes [16,17,18]. Compared to the all-inorganic Cs- or organic–inorganic CH_3_NH_3_-based PNPs, the organic–inorganic formamidinium-based PNPs have higher stability such as higher chemical, thermal, and moisture stability [15,19,20,21,22,23,24]. Nevertheless, the poor stability of organic–inorganic hybrid perovskites against oxygen, water, and thermal treatment has restricted their actual applications [25].

Several methods were presented to improve the stability of the PNPs. For example, enclosing PNPs in poly (methyl methacrylate) [26,27], polyhedral oligomeric silsesquioxane (POSS) [28] and inorganic SiO_2_ network structure were used to effectively keep optical and chemical stabilities of the PNPs. Compared with the organic encapsulation coating, the inorganic SiO_2_ encapsulation was widely used to prevent the influence of atmospheric moisture and oxygen for PNPs [29,30]. In addition, silica-wrapped PNPs could be applied in phosphor powders and light conversion films to exchange the light-emitting color. Hu et al., reported the silica-coated process to encapsulate CdSe/ZnS QDs in 2009 [31], but silica-coated CsPbX_3_ (X = Cl, Br and I) PNPs compounds were fabricated until 2016 [32,33]. Subsequently, APTES [34,35,36], tetraethylorthosilicate (TEOS), and Tetramethoxysilane (TMOS) were utilized to form silica-coated CsPbX_3_ (X = Cl, Br and I) PNPs.

The PNPs were synthesized by a typical hot injection process and a post treatment for encapsulation, which exhibits a low throughput. Sun and coworkers used a one-pot method to prepare silica-coated CsPbX_3_ (X = Cl, Br and I) PNP, which added a little number of APTES during the hot injection process. This is an easy and effective method to improve stability [34]. Organic–inorganic CH_3_NH_3_PbBr_3_ PNPs were also prepared in a facile room-temperature one-pot method employing (3-aminopropyl) trimethoxysilane (APTMS) [37], which ensures high luminescence and stability using an easy and rapid strategy. It is highly desirous to develop a near-infrared (NIR) light for the tracking of muscle or disabled patients in the detection of blood vessel, because 650–950 nm wavelengths in NIR are less significantly absorbed by human skin, and can therefore penetrate deeper into the body [38]. Therefore, a one-pot method is necessary for silica-wrapped NIR FAPbI_3_ PNPs at room temperature in open air.

Herein, a fast, simple, and efficiency strategy to synthesize high-stability PNPs embedded into silica by air synthesis at room temperature was demonstrated. The perovskite nanocomposites (PNCs) were prepared via a APTES hydrolysis encapsulation strategy. The NIR PNCs was very stable in several rigorous conditions, such as storing in the humid air and ultrasonication in water. In addition, NIR-LED devices were also prepared by FAPbI_3_ PNCs as the light-conversion materials coated on the commercial blue InGaN chip. The PNCs exhibits well moisture-resistant and air stability with a long operating lifetime compared to FAPbI_3_ PNPs.

## 2. Materials and Methods

### 2.1. Air-Synthesis of NIR-FAPbI_3_ PNPs and PNCs

First, 0.1 mmol of formamidine acetate (99%) was dissolved in 10 mL OCTA and stirred 10 min at room temperature (25 °C) in open air for preparation FA precursor as the first step. Then, 0.1 mmol of lead (II) iodide (PbI_2_, 99.999%) were dissolved in a mixture of 10 mL of toluene (98%), 0.8 mL of oleic acid (OA, 90%), 1.2 mL of oleylamine (OAM, 90%), and 1 mL of APTES (99%) at room temperature in the air under stirring for 1 h until PbI_2_ was completely dissolved. Subsequently, 2 mL of FA precursor solution was added into the mixture and vigorously stirred for 30 min. The mixture solution was added to hexane (95%) and centrifuged at 9000 rpm for 5 min and the hexane was used to disperse the precipitates. After the second centrifugation, the powders of the NIR-FAPbI_3_ PNCs can be obtained by removing the hexane under the airflow at room temperature.

### 2.2. Manufacture of NIR-LEDs and Characterization

The NIR-FAPbI_3_ NCs powders and the UV resin (weight ratio = 1:2) were mixed, coated on blue LED chips (wavelength = 455 nm), and baked at 70 °C for 5 min in an oven. Consequently, UV curing for 30 s in air used a 365 nm UV lamp to obtain the color-converted layers. Electroluminescence (EL) performances were measured using an LQ-100R spectrometer (Enlitech, Kaohsiung, Taiwan). Photoluminescence quantum yield (PLQY) and photoluminescence (PL) were obtained using F-7000 (Hitachi, Tokyo, Japan). The surface morphologies of samples were observed using JEM-2100 (JEOL, Tokyo, Japan) and JSM-7610F (JEOL). FTIR spectra was measured using spectrum one (PerkinElner, Waltham, MA, USA). X-ray diffractometer (XRD) patterns were measured using a D8 ADVANCE (Bruker, Billerica, MA, USA).

## 3. Results and Discussion

**Figure 1:**
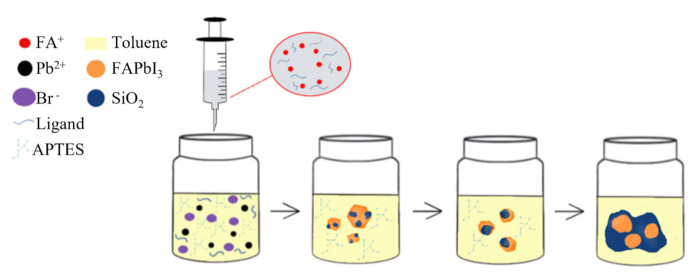
Schematic of the air-synthesis method for preparation of FAPbI_3_ NCs.

PNCs were obtained through the air synthesis at room temperature. The simple reaction system, PbI_2_, OA, OAM, toluene, and APTES in one pot, was stirred 30 min at room temperature (28 °C) in open air (Figure 1). The FA precursor was then rapidly injected into the mixture, and the colorless solution turned dark red immediately, which indicates the constitution of FAPbI_3_ PNCs (Video S1, Supporting Information).

**Figure 2:**
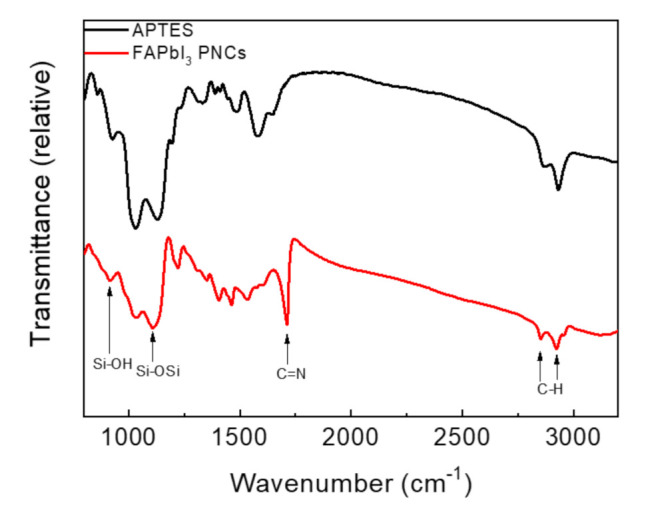
FTIR spectra of APTES and FAPbI_3_ NCs.

The APTES molecule provides Si–O bonds which generate the Si–O–Si ligands through hydrolysis and dehydration in the reaction to package PNPs. This protects PNPs from environmental factors [39,40,41]. Therefore, to verify Si–O–Si ligands on the surface of PNCs, a FTIR spectrum was used to prove the silica wrapping (Figure 2). The absorption peak at 914 and 1108 cm^−1^ can be observed in the FAPbI_3_ PNCs sample, which is attributed to Si–OH bonds caused by the hydrolysis condensation of APTES and asymmetrical Si-O-Si groups, respectively. These two peaks at 914 and 1108 cm^−1^ indicate that APTES is well bonded to FAPbI_3_ PNCs. In addition, there is a strong stretching vibration at 1710 cm^−1^ due to C=N from FA^+^. The C–H stretching vibrations of CH_2_ and CH_3_ were detected from 2800 to 3000 cm^−1^ [41,42,43]. 

**Figure 3:**
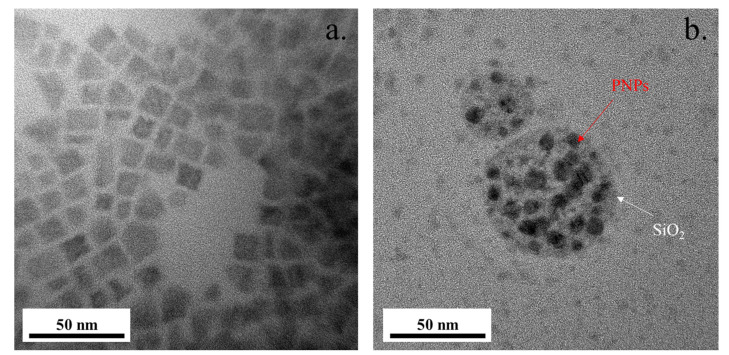
HRTEM images of (**a**) PNPs and (**b**) PNCs.

In order to verify that the PNPs embedded in silica, and confirm the real PNCs structure, the morphological features of the PNCs were observed by TEM. HRTEM images (Figure 3a) show that the as-synthesized FAPbI_3_ PNPs have a cubic shape. Figure 3b shows the HRTEM image of the as-prepared FAPbI_3_ PNCs; the PNPs embedded into a shapeless material can be clearly seen, which suggests the presence of SiO_2_ materials. These SiO_2_ shells protect the PNPs from the influence of atmospheric moisture and oxygen [29,30]. The particle sizes have provided in Figure S1. Si and O elements can be detected by Energy dispersive spectroscopy (EDS) of Figure 3b (Figure S2), which is the evidence for the silica presence. The particle sizes of PNPs and PNCs were established to be 16.8 and 10.6 nm, respectively. The smaller size of PNCs may be due to the fact that the Si–O–Si ligands inhibit contacts between FAPbI_3_, leading to limited particle growth. Similar results were observed in X-ray diffraction (XRD) patterns, as shown in Figure S3. Both samples only showed the cubic phase of FAPbI_3,_ indicating amorphous SiO2. Compared with PNPs, PNCs exhibited weaker XRD intensity, which was attributed to smaller particle size and lower perovskite particle density in the powder. Meanwhile, compared with air, the higher refractive index of SiO_2_ can enhance the light extraction from PNCs. 

**Figure 4:**
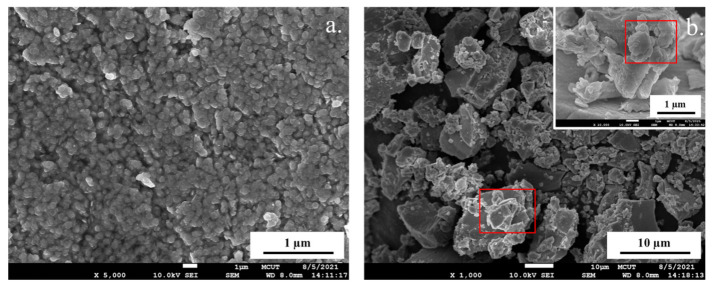
FESEM images of (**a**) PNP and (**b**) PNC powders.

Figure 4 shows the FESEM images of the PNPs and the PNCs powders. Figure 4a shows that the larger grain size (approximately a few hundred nanometers) in the PNP powders is much greater than the TEM observation, which indicates that the PNPs aggregate without SiO_2_ protection. The larger particles in Figure 4b were attributed to the SiO_2_ matrix growth and network covalent solid of SiO_2_. Thus, the abovementioned results evidence that the PNPs and PNCs can be obtained using our simple room-temperature synthesis method. 

**Figure 5:**
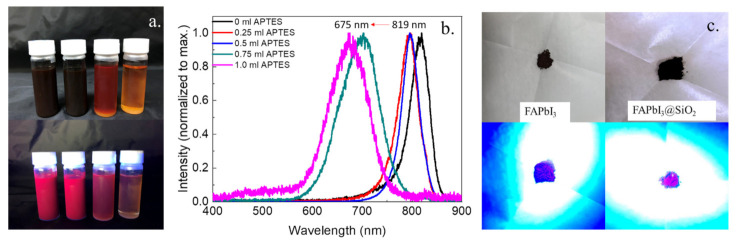
(**a**) Photographs of FAPbI_3_ NCs solvent (from left to right: 0.25–1.0 mL of APTES) under room light and the UV light respectively; (**b**) the PL spectra of FAPbI_3_ NCs with different amount of APTES, and (**c**) photographs of FAPbI_3_ and FAPbI_3_NCs powders under room light and UV light.

The PL spectrum of 0.25 mL APTES exhibits a narrow symmetric emission band with a peak at 795 nm, with a longer wavelength because of the scattering effect of large particles, as shown in Figure 5. However, an inadequate number of ligands leads to low PLQY (ca. 23%). When the APTES concentration increases to 0.5 mL, the highest PLQY (58.0 %) was obtained with a slight blue-shift emission. Although the emission could be further blue-shifted, the PLQY of NCs reduced. It is known that with high ligand concentrations, the rate of the reactive molecules’ delivery through the silica-wrapped layer becomes slower due to the steric hindrance of Si–O–Si, resulting in smaller particles and the reduced PLQY [38]. Figure 5c shows the as-prepared PNPs and PNCs powders.

**Figure 6:**
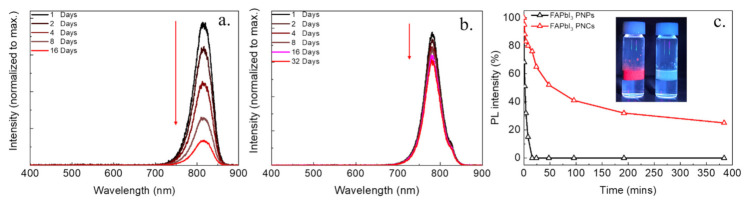
The PL spectra of FAPbI_3_ (**a**) PNP and (**b**) PNC powders stored in air after different days; (**c**) the intensity of the PL peaks under the DI water as a function of times for FAPbI_3_ NPN and PNC-dispersed solutions. The insets show the photographs of the FAPbI_3_ NPNs and PNCs added into water after 16 min.

To confirm that PNCs effectively blocks moisture and oxygen in the atmosphere, the PL spectra of the respective powders stored at approximately 26 °C with a relative humidity of approximately 75 % were measured for the different storage times. The PL intensities of the FAPbI_3_ PNPs showed an obvious decay after 16 days, which is in agreement with previous reports [34,39,43], as shown in Figure 6a. In contrast, Figure 6b exhibits a slow decrease in PL intensity which suggests a good stability in the moist air for the FAPbI_3_ PNCs. Furthermore, the water stability of FAPbI_3_ PNCs was recorded by 1 mL of FAPbI_3_ PNP and PNC solutions injecting to 2 mL of DI water. Figure 6c shows the PL intensities of FAPbI_3_ PNP and PNC solutions in DI water; the dark red fluorescence of FAPbI_3_ PNPs solution decayed swiftly after 16 min in DI water. However, the FAPbI_3_ PNC solution still showed dark red light in the DI water even after 32 min, as shown in Figure 6c. It also remained 25% of initial PL intensity after 384 min. In contrast, the FAPbI_3_ PNCs, revealed better water stability for the FAPbI_3_ PNCs.

**Figure 7:**
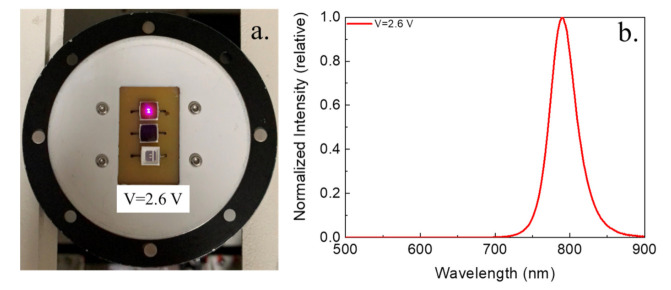
The photographs of the blue chip (455 nm) and the blue chip consisting of FAPbI_3_NCs under the (**a**) room light and (**b**) the NIR-LED devices EL spectrum.

The NIR FAPbI_3_ PNCs powder was coated on blue InGaN chip (wavelength = 455 nm) and NIR-LEDs were fabricated, as displayed in Figure 7a. Figure 7b shows a typical EL spectrum of NIR LEDs located at 788 nm under 2.6 V, indicating NIR emission. This may have a potential as a NIR light source to detect a blood vessel. Our results indicate that moisture-resistant and air-stability FAPbI_3_ PNCs synthesis at room temperature is a promising material in bio-optoelectronic devices.

## 4. Conclusions

In conclusion, we successfully synthesized FAPbI_3_ embedded into silica at room temperature in open air by a facile method. The air-synthesized PNCs at room temperature treatments still display high stability under ambient exposure and a narrow emission in the PL spectra. In particular, the SiO_2_ protective layer provides high PL intensity after 32 days of storage atmosphere (28 °C) and stability in DI water. The NIR-LEDs based on the NIR-emitting FAPbI_3_ PNCs powder coated on the blue LED have a 788 nm EL spectra. We hope our results can be further applied in biomedical lighting applications and devices based.

## Supplementary Materials

The following are available online at https://www.mdpi.com/article/10.3390/bios11110440/s1, Figure S1: The corresponding particle sizes of Figure 3 for PNPs and PNCs; Figure S2: Energy dispersive spectroscopy (EDS) of Figure 3b; Figure S3: X-ray diffractometer (XRD) patterns of FAPbI_3_ PNP and PNC powders; Figure S4: PL intensities of the studied PNCs under (a) UV radiation (365 nm; ~0.1 W/cm^2^) and (b) heating treatment (100 °C) for different times.
