# Supplementary material for: Room-Temperature Synthesis of Air-Stable Near-Infrared Emission in FAPbI3 Nanoparticles Embedded in Silica
Source: Biosensors (Basel). 2021 Nov 4;11(11):440. doi: 10.3390/bios11110440 (PMC8615587; doi:10.3390/bios11110440)
Supplement: Supplementary file 1 [file biosensors-11-00440-s001.zip › biosensors-1399804-supplementary.pdf]

biosensors

Supporting information

Room-Temperature Synthesis of Air-Stable Near-Infrared Emission in $FAPbI_3$ Nanoparticles Embedded in Silica

Lung-Chien Chen [1], Li-Wei Chao [1], Chen-Yu Xu [2], Chih-Hung Hsu [3], Yi-Ting Lee [4], Zi-Min Xu [1], Chun-Cheng Lin [5,*] and Zong-Liang Tseng [2,*]

[1] Department of Electro-optical Engineering, National Taipei University of Technology, Taipei 106344, Taiwan; ocean@ntut.edu.tw (L.-C.C.); aa0932693328@gmail.com (L.-W.C.); eok26732687@gmail.com (Z.-M.X.)
[2] Department of Electronic Engineering, Ming Chi University of Technology, New Taipei City 24301, Taiwan; u06157020@mail2.mcut.edu.tw
[3] Giant-Tek Corporation, Miaoli County 35048, Taiwan; rex@giant-tex.com.tw
[4] Center for Organic Photonics and Electronics Research (OPERA) Kyushu University 744 Motooka, Nishi, Fukuoka 819-0395, Japan; ytlee@opera.kyushu-u.ac.jp
[5] Department of Mathematic and Physical Sciences, General Education, R.O.C. Air Force Academy, Kaohsiung 82047, Taiwan
* Correspondence: cclincafa@gmail.com (C.-C.L.); zltseng@mail.mcut.edu.tw (Z.-L.T.)

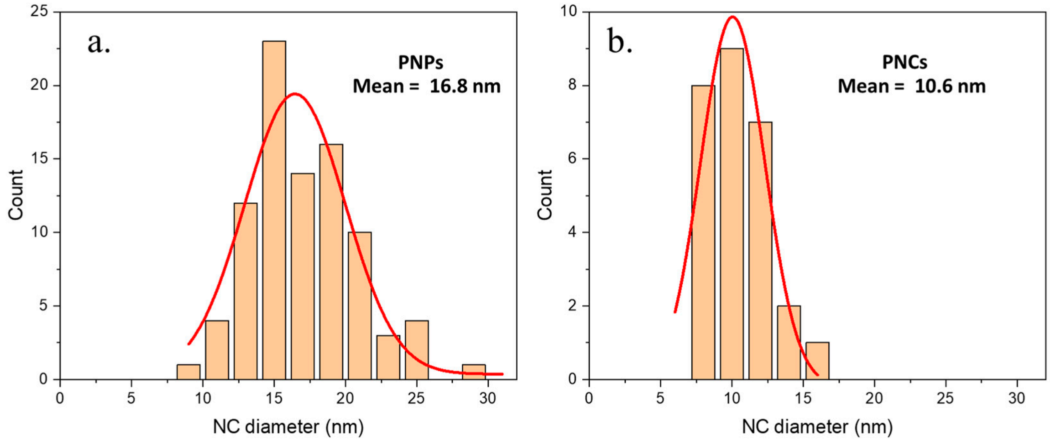

Figure S1. The corresponding particle sizes of Figure 3 for PNPs and PNCs.

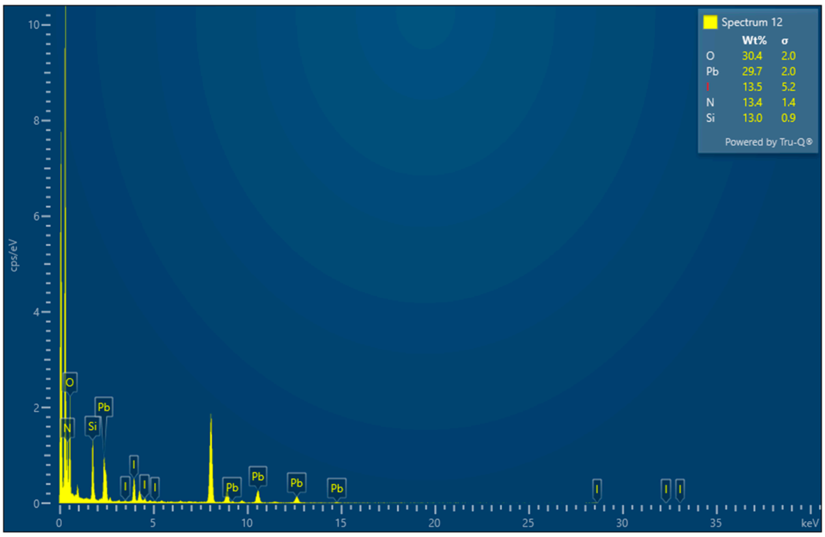

Figure S2. Energy dispersive spectroscopy (EDS) of Figure 3 (b).

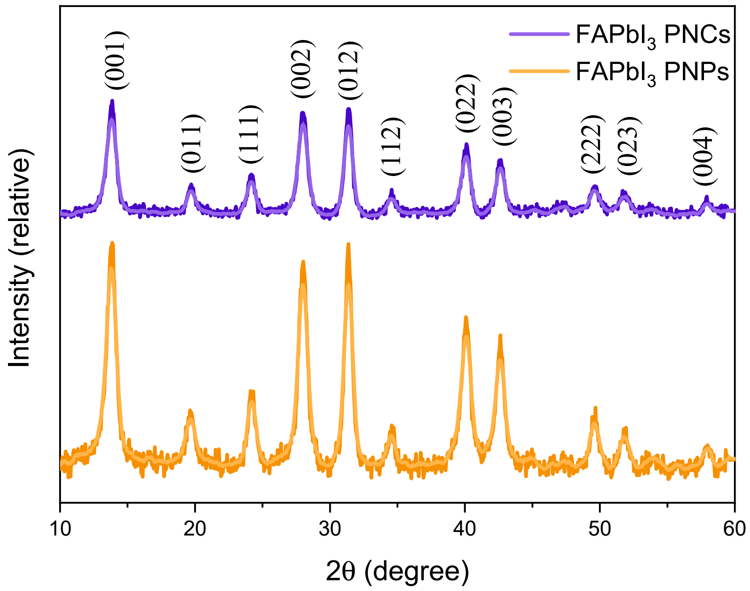

Figure S3. X-ray diffractometer (XRD) patterns of $FAPbI_3$ PNP and PNC powders.

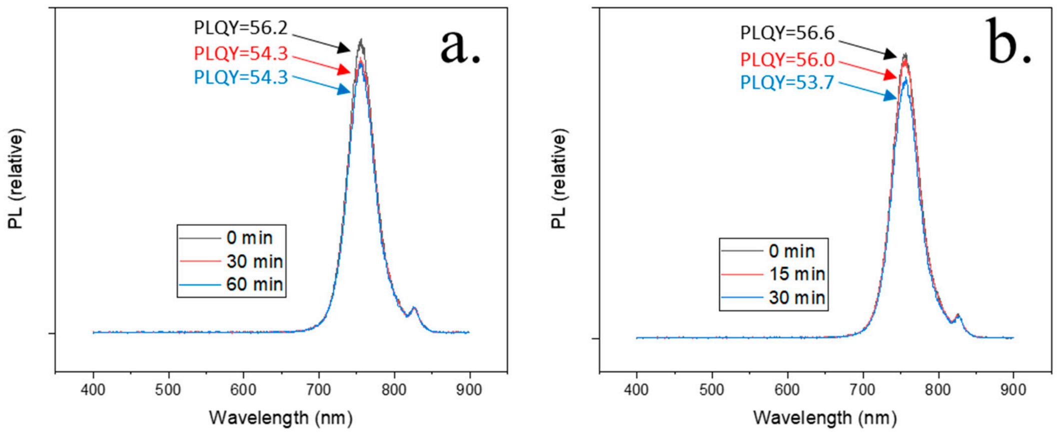

Figure S4. PL intensities of the studied PNCs under (**a**) UV radiation (365 nm; ~0.1W/cm^2) and (**b**) heating treatment (100 ^{o}C) for different times.
